# Digital Marketing Strategies for Sustainable Food and Beverage Brands: A Bibliometric Systematic Literature Review

**DOI:** 10.12688/f1000research.176435.2

**Published:** 2026-02-20

**Authors:** Vishal Soodan, Supernova Chakraborty, R K Tailor, Deeksha Sharma

**Affiliations:** 1 Department of Commerce, Manipal Academy of Higher Education, Manipal, Karnataka, India; 2 Center for Distance and Online Education, Manipal University Jaipur, Jaipur, Rajasthan, India; 3 Chhatrapati Shahu Institute of Business Education and Research Trust, Kolhapur, Maharashtra, India; 4 International Academy of Management and Entrepreneurship, Bengaluru, Karnataka, India

**Keywords:** Digital Marketing, Food and Beverages, Social Media Analytics, Sustainability

## Abstract

Digital Marketing is one of the most important channels for promoting products and services in the modern age of marketing. It leverages popular tools, including social and web-based resources, to promote a brand in an online space. As consumers become aware of environmental degradation, researchers and academicians are immensely capturing consumers’ responses towards brands that minimize environmental impact. The present study is an attempt to provide a bibliometric account of research on Digital Marketing and Sustainable Food and Beverage Brands from the Scopus database. Based on 53 articles filtered through well-defined inclusion and exclusion criteria using B-SLR, the study analysed the conceptual structure of the work published between 2014-2024. This research uncovers emerging research streams and highlights the overlooked nuances of SDM in Food and Beverage brands. The review further provides strategic insights for firms that actively engage customers through digital media.

## 1. Introduction

A continuous increase in the global population increases the risk of environmental damage to the environment. ^1^ Rising demand levels are a key contributing factor to the carbon footprints. ^2, 3^ In response, organizations providing products and services are also under growing pressure to shift their strategies to accommodate the evolving expectations of diverse stakeholders while taking care of society’s long-term interests. This strategic realignment has strengthened the demand to adopt and uphold sustainable practices. Moreover, consumers are increasingly drawing on green and sustainable offerings. Research indicates that products and brands incorporating sustainability claims exhibit stronger growth trajectories than their conventional counterparts. ^4^ Given the increasing importance of sustainability and growing concerns over environmental issues, there is an urgent need to adopt credible and environmentally friendly practices to address these challenges effectively.

Governments worldwide have emphasized the importance of promoting environmentally friendly products to support green marketing. Strict environmental policies have been adopted to minimize sustainability issues related to the sustainability. ^5^ An urgent need is for environment-friendly innovations, including products, services, and business models that aim to reduce the overall impact on natural resources. ^6^ Previous research on green products and sustainability has typically adopted a broad approach, examining various product categories and services in the far-reaching domain of green marketing. ^7– 9^ However, it is important to understand that consumer behavior and marketing strategies often differ significantly across product types. For food products, the role and impact of sustainability may differ from those observed in other categories. Thus, it is essential to explore the sustainability of food products and their development over the past decade (2014-2024), particularly through digital marketing.

The rapid development and spread of digital technology marketing presence has led to a revolution in the marketplace. Marketing is now mainly centred on digital media such as blogs, websites, email, and social networks. ^10^ However, digital marketing extends far beyond traditional internet marketing. Modern digital tools encompass a mix of online marketing tools digital technologies, including websites, email, databases, and interactive platforms such as blogs, feeds, podcasts, and social networks. The growing adoption of digital technology has significantly influenced consumer behavior, reshaping how people make purchasing decisions. ^11^ Consequently, marketers must understand and adapt to the impact of digital marketing. Effective digital marketing requires well-thought strategies and selection of appropriate media to engage target audiences, increase sales, and drive profitability. In addition, with the rapid growth of D2C brands, the traditional e-commerce model has witnessed rapid growth by bypassing traditional retailers and distributors. Additionally, the adoption of the omni-channel approach has further helped the growth of online food sales.

Digital marketing strategies for sustainability in F&B require bibliometric examination due its consumer-centric characteristics that differentiate it from a general branding or digital marketing contexts related to sustainability. Additionally, sustainability in the F&B is directly associated with emerging issues such as food safety, ethical sourcing, environmental impact, sustainable packaging and health, which significantly induce consumer trust and purchase decisions. Digital platforms play a crucial role in sustainability through transparency, traceability, and credibility. Hence, the sector-specific dynamics affect research themes, author collaboration patterns, and knowledge structures that cannot be captured when investigated under a broader perspective. Therefore, a bibliometric analysis is required to map intellectual structure, emerging trends, and thematic evolution in F&B domain thereby providing more precise theoretical and managerial insights.

Previous studies have attempted to identify the factors responsible for consumers’ adoption of green or sustainable food. A review of literature reveals the relationship between green marketing strategies and the resulting consumer behavior in different contexts. A study on green marketing identified the major themes of green purchases and green marketing mix using thematic analysis. ^12^ More recently, a comprehensive assessment of peer-reviewed empirical studies was conducted to gain insights into the interaction between green marketing strategies for sustainable food and consumer behaviour ^13^ findings indicate the role of digital marketing in establishing an effective relationship among players in an agri-food system. ^14^ An increase in the diffusion of digital technologies has significantly influenced consumer purchasing decisions, ^11^ triggering a plethora of research covering the broad domain of food and beverages. ^15, 16^ Researchers have attempted to uncover the role of digital advertisements, ^17^ media content and peer influence, ^18^ digital marketing techniques, ^19, 20^ brand partnerships ^21^ and influencers in the marketing of food and beverages. ^22^ Scholars have outlined the darker side of digital marketing in the industry, providing deeper insights into policy-related challenges, ^23^ vulnerabilities in the use of social media ^24^ and overexposure. ^25^ However, to our knowledge, no previous study has highlighted digital marketing strategies for sustainable food and beverage brands through a review of the literature from the past decade. As these brands are gaining prominence in the digital sphere, there is an urgent need to explore the studies conducted in the past decade (2014-2024). Furthermore, previous studies have focused on the customer perspective, and no attempt has been made to underline strategic perspectives related to sustainable food and beverage brands in an online ecosystem.

Thus, as evident from the literature, no previous review has captured an inclusive understanding of digital marketing strategies for sustainable food and beverage (F&B) brands. Consequently, this study attempts to address this issue by analyzing and integrating the relevant literature on sustainable foods and digital/online markets/retail in recent years. This study aims to address the following research questions (RQs):

RQ1 : How has research on digital marketing strategies for sustainable food and beverages (F&B) evolved over time, and what theoretical domains do it draw upon?

RQ2 : What thematic clusters emerge and how do they contribute to building a conceptual framework for sustainable digital branding?

RQ3 : Where are the gray areas, and how can future research advance theory and practice in sustainable digital marketing?

To address these research questions, we developed a systematic literature review (SLR) based on the 5W1H pattern. ^26^ The pattern was selected because it is the most appropriate method to map out the state of research on a topic. ^27^ This method is widely implemented across various contexts for frame analysis in literature reviews. ^28^ 5W1H represents what, who, where, when, how, and why utilizing this pattern, we attempted to answer the following questions.

What: What are the key findings of this literature review?

Who: Who are the most active researchers? (Collaborations, Affiliations)

Where: Areas of the world where the research is widely published.

When: The span of time for which research has been conducted.

How: How the research in this domain will add to the existing information?

Why is it important for future researchers to choose this area for future research?

### 1.1 Sustainable food

The concept of sustainable food involves methods that curtail environmental footprints and increase food safety and quality. ^29^ This implies that food needs to be produced and moved in a way that does not emit greenhouse gases, deplete resources, and avoids over farming. Sustainability also means cutting down on food waste, production costs, and producing food that is healthy and profitable. ^30, 31^ As environmental issues are the primary focus, human rights, working conditions, and animal welfare are also taken into consideration. ^32^ The goal is related to sustainable consumption; however, studies have found contradictory findings. For example, some studies ^33, 34^ showed that people don’t usually choose what they eat because they want to help the environment or animals. ^35^ This shows that sustainable food purchasing habits are still not well understood. It is especially true in places where people are just now becoming more aware of their environment. ^36^ Factors such as habit, price, health benefits, enjoyment, and subjective standards determine the amount of food consumed. ^37, 38^ From a commercial perspective, sustainable food retailing has been revolutionized by digital marketing. With the emergence of new channels, brand marketing has transformed the way businesses communicate with consumers. ^39^ The F&B industry also leverages digital wave by providing improved access and reducing costs. This disruption in the marketplace opens opportunities and challenges for marketers, researchers, and policymakers of strategic marketing in the age of digital transformation.

### 1.2 The concept of sustainability in the food industry

The concept of sustainability in the food industry is contentious, even compared to other sectors. The term ‘sustainable food’ is extensive and challenging to define for both consumers and policy makers. ^40^ The term “sustainability” was employed to support the principles of sustainable growth. In 1992, the United Nations Conference on Environment and Development (UNCED) incorporated the economic and social dimensions. Subsequently, the three pillars of sustainability currently garnered increased attention and received endorsement from multiple authors worldwide. Therefore, sustainability in the food industry covers practices and priorities that businesses can employ. As one of the largest contributors to greenhouse gas emissions, there is scope for improvement and further research in this domain.

### 1.3 Digital marketing and food industry

Digital technology is undoubtedly one of the most remarkable innovations, especially when assessing value based on its ability to enhance people’s lives. ^41^ Over the past 25 years, the rapid emergence of the Internet and evolution of modern information and communication technologies have completely transformed traditional marketing principles. This change has brought a new era known as “digital marketing,” driven by the rapid expansion of media and globalization of the market. ^42^

Today, marketers understand the advantages of digital marketing, including accessibility, control, simplicity, and cost-effectiveness. Empirical studies also confirm the connection between digital brand communication and essential consumer attributes such as trust and loyalty. ^43, 44^ Emerging technologies like artificial intelligence, the Internet of Things, smart devices, and machine learning are already shaping consumer behaviour and as well as consumer experiences. ^38^ In contrast to traditional marketing, digital marketing serves as a communicator between business and customers. The ability to customize marketing activities has been improved by providing consumers with more control and allowing them to co-create value in their experiences, especially in reference to emerging social behavior. ^37^

Digital platforms help to establish relationships between companies and customers. Platforms that support digital presence, encourage interaction, and drive engagement serve as the foundation for this evolving digital entity. ^45^ Digital marketing includes a wide range of approaches such as banner ads, digital advertisements, sponsored social media content, and other interactive marketing strategies. The interactive and engaging nature of digital marketing differentiates it from traditional marketing, which is a targeted, segmented, and measurable perspective. ^46^ Every digital marketing campaign has a particular set of audiences, clear objectives, and measurable key performance indicators (KPIs) . ^47^ Digital marketing is an affordable alternative, as it may improve marketing strategies and reach a targeted set of customers to earn the best value. When strategically employed, it is simple to incorporate into a company’s marketing plan. The market becomes more open, and it also makes business easier to function. This leads to better experiences for both businesses and customers. ^48^

Digital marketing has introduced new opportunities and challenges for both researchers and professionals. Digital marketers must stay updated with rapidly evolving tools and technologies for efficient performance. Handling vast amounts of data requires comprehensive analysis, which is a challenge. Many businesses face issues when integrating digital strategies into existing business models. A good understanding of consumer behavior and purchasing intention is important for successful digital marketing campaigns. Over the last decade, it has been witnessed that more than half of the world’s population actively uses social media, which has become an essential part of daily life. ^49^ The success of digital marketing strategies depends on the companies’ need to set specific goals aligned with customer expectations, feedback, and technological advancements to be relevant in business. ^50^ Digital marketing may help food and beverage brands develop multichannel marketing and advertising strategies, which can help achieve better target markets, brand awareness, customer engagement, and profitability. The capabilities of the Internet and digital technology establish a virtual marketplace leading to digital transformation of sectors, thereby enabling wider reach, better conversion rates, and a strong digital presence. ^51^

## 2. Methodology

The research intends to answer the questions by employing the Bibliometric Systematic literature review (B-SLR) framework. ^52^ This study limits the review to the period of 2014-2024 to get insights into the trend and recognize the increasing awareness of consumers towards sustainable F&B. This decade was selected for the study, as research production related to sustainable consumer behavior saw an increase after the UN defined the ‘Sustainable Development Goals’(SDGs) in 2015. ^13^ In the context of digital marketing, this period is considered promising for e-commerce. ^53^ In the year 2014, Deloitte reported that the top 250 online retailers registered a growth of 25% for the first time ever. ^54^ Furthermore, in 2014, worldwide retail sales exceeded $1 trillion and witnessed a growth rate of 19.4%. In addition, we followed the suggestions given by well validated study findings, ^55^ advocating the selection of literature from the year of emergence as an inclusion criterion. Therefore, keeping the above-mentioned suggestions in mind, 2014 was selected as the base year for this review.

The present study applies the bibliometric systematic literature review (B-SLR) framework. ^52^ The B-SLR method was used because it is well-suited to the context of management studies and can be applied where literature reviews require a detailed examination of a specific research domain. ^56^ Another reason for employing this method was to outline and synthesize existing literature in the domain of sustainable digital marketing and to position new contributions. First, we scrutinized the literature on sustainable foods, beverages, and digital marketing to build an overview of the research stream and implement a set of representative keywords for database research. Following the B-SLR framework, we outlined our inclusion and exclusion criteria before approaching the data-collection process. To do this, we leveraged a comprehensive set of key definitions in the field of inquiry. ^55, 57^ We employed PRISMA as the reporting guideline to identify, select, appraise, and synthesize studies from the literature. ^58^ PRISMA was used to narrow down the set of studies that were less relevant to the review and to increase transparency. ^59^We defined the search query as follows: (TITLE-ABS-KEY(“online sales” OR “digital marketing” OR “online marketing” OR “internet marketing” OR “social media marketing” OR “e-commerce” OR “mobile commerce” OR “quick commerce”))AND (TITLE-ABS-KEY (“environmental impact” OR “carbon footprint” OR “sustainable food” OR “sustainable beverage” OR “sustainable drinks” OR “eco-friendly food” OR “green food” OR “organic food” OR “environmental labelling” OR “Ecolabels” OR “sustainable food production”)). A multi-stage inclusion and exclusion process was used to refine the sample studies following a well-validated set of criteria. ^60^

To qualify, articles had to be included in the Scopus database. This database was selected for the study, as it is the largest existing multidisciplinary database for research. There are approximately 27,950 peer-reviewed journals indexed in Scopus. ^57^ The quality and peer review in Scopus provide a solid foundation for research. Another reason for selecting Scopus over the Web of Science is the coverage of content. ^61^ In addition, Scopus is known for its reliability and reduced risk of bias. ^62^ Google Scholar was used to reduce location bias, and Google search was employed to retrieve reports and official publications.

Journal articles should be peer-reviewed, and the language of publication must be English, with full text available. In total, we retrieved 266 articles from the Scope database and 21 articles from Google Scholar and Google searches. After removing the duplicates, screening resulted in obtaining 271 articles.

We further refined the dataset after cross-validating the search results by removing ineligible entries. Additionally, we screened the raw dataset to exclude studies that did not match the description of consumption behavior and strategies promoting ethical food purchases as an outcome. This resulted in 204 entries being removed.

Of the 67 full-text articles accessed for eligibility, we removed 14 articles based on their findings, which were not relevant to the research question. As inclusion criteria, we also included articles involving literature reviews (03), case-based evidence (07), and text mining (01), thereby totalling the total and final number of articles to 53. Sensitivity analyses were performed by employing leave-one-out analysis, a method that is well known for its robustness. ^63^ We evaluated the impact of excluding studies with a high Risk of Bias on the robustness of the results. We also evaluated the impact of excluding studies that did not consider clustering.

**Figure 1.**
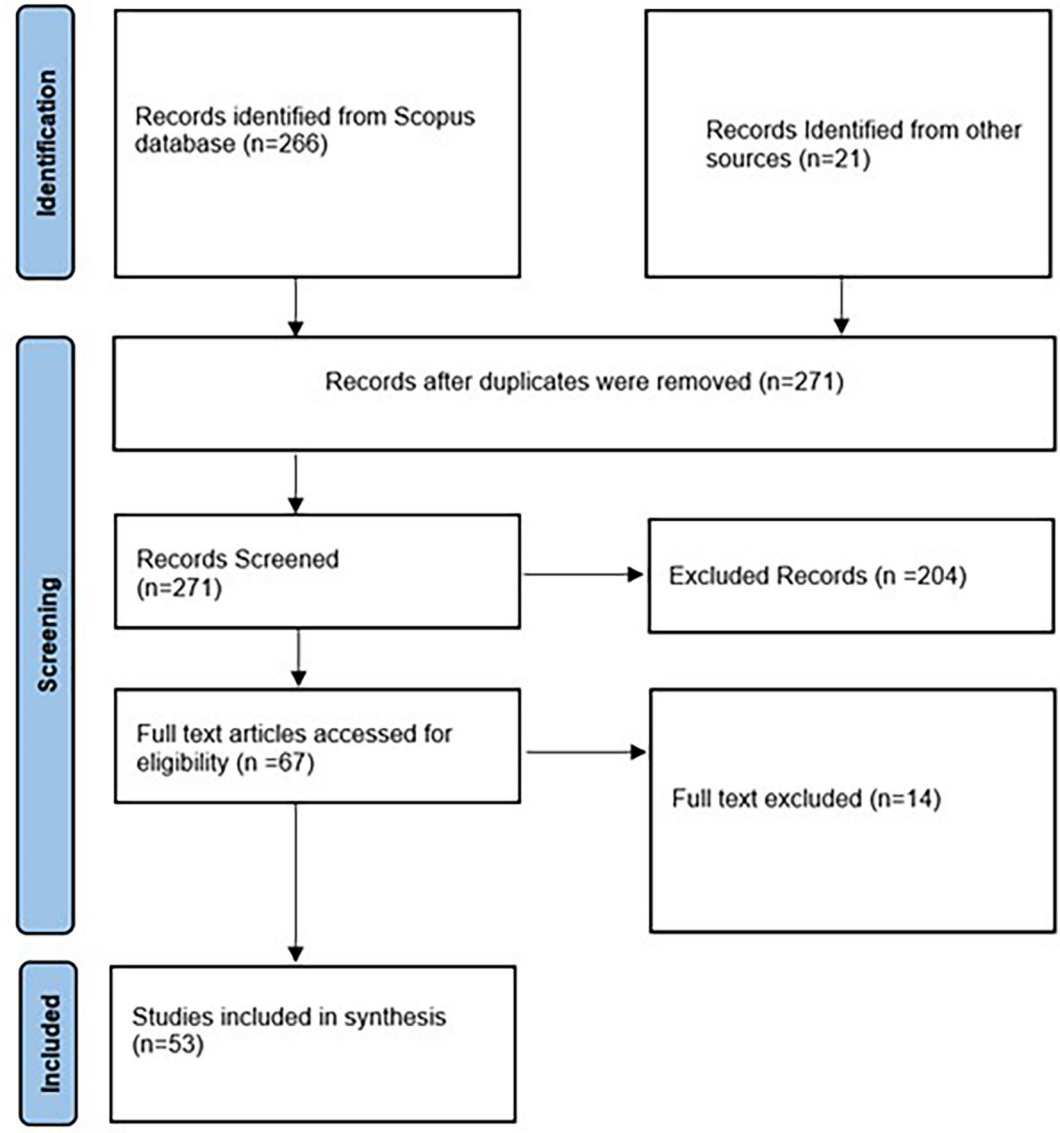
Prisma flowchart (Page et al., 2021).

Next, each author independently carried out the screening process by assessing the inclusion and exclusion of Krippendorff’s alpha coefficient. ^64^ Before the rating exercise, the authors underwent a training session that included familiarizing them with the rating scheme, practising rating exercises, and discussion to clarify any ambiguities. The confidence intervals were determined at the 95% level with 1000 bootstrap iterations. The rated data were then input into the web-based statistical package, K-Alpha Calculator. The analysis provided a reliability coefficient for the coding scheme, indicating the extent of agreement among raters. The resulting Krippendorff’s alpha coefficient was 0.775, indicated the desired result for the study. ^52^ Figure 1 depicts the PRISMA 2020 flowchart that illustrates the search procedure.

To ensure that the exclusion criteria for databases did not result in bias, we followed the recommendations outlined in the literature. ^65– 67^ Bias related to the review design was addressed through a comprehensive inclusion and exclusion process and by calculating Krippendroff’s alpha. ^52^ Location bias was addressed using specific filters. However, issues related to selection bias were reduced by removing ambiguity. ^66^ The extracted documents were read independently to obtain key research topics within each cluster. Disagreements between the authors for the final set of studies were resolved by detailed discussion and developing a consensus with reasons for final decisions of exclusion at full text recorded against citations. ^68^ Once a satisfactory level of agreement among the authors was reached, the minimum cluster size was determined (n = 4). We assessed and ranked the documents based on their representation to form four clusters: 1. Brand Impact 2. Brand Logistics 3. Brand Identity 4. Brand Economics. The final dataset was used to perform a bibliometric analysis using the bibliometrix package in R Studio ( https://www.bibliometrix.org).

## 3. Results and discussion

### 3.1 Annual scientific production of articles

**Figure 2.**
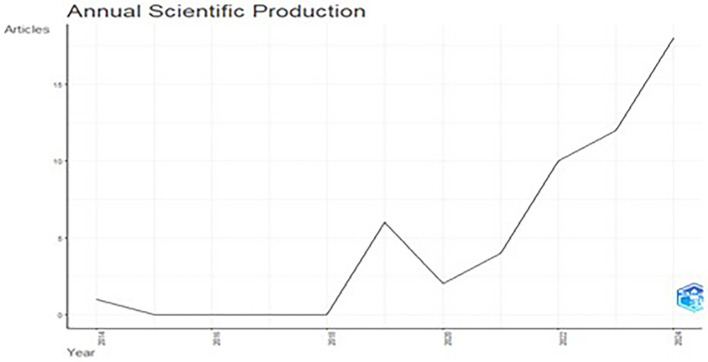
Annual scientific production.

Figure 2 illustrates the yearly distribution of articles published from the year 2014-2024. The data revealed consistency in scientific output from 2015 to 2016, followed by a substantial rise in publications from 2018 onward, with a sharp peak observed in 2019 ( Figure 2). This trend indicates growing research activity, as these years saw the immense popularity of e-commerce platforms across the world. Interestingly, 2019 saw a decline and the post 2020 annual article again saw a rise. From 2020 onward, there was a clear upward trajectory in scientific output, with a sharp increase by 2023. With an upward trend for 2024, this suggests an expanding interest and investment in the field, with a growing number of researchers contributing to the body of knowledge, particularly in the last decade.

### 3.2 Keywords distribution (Tree map)

**Figure 3.**
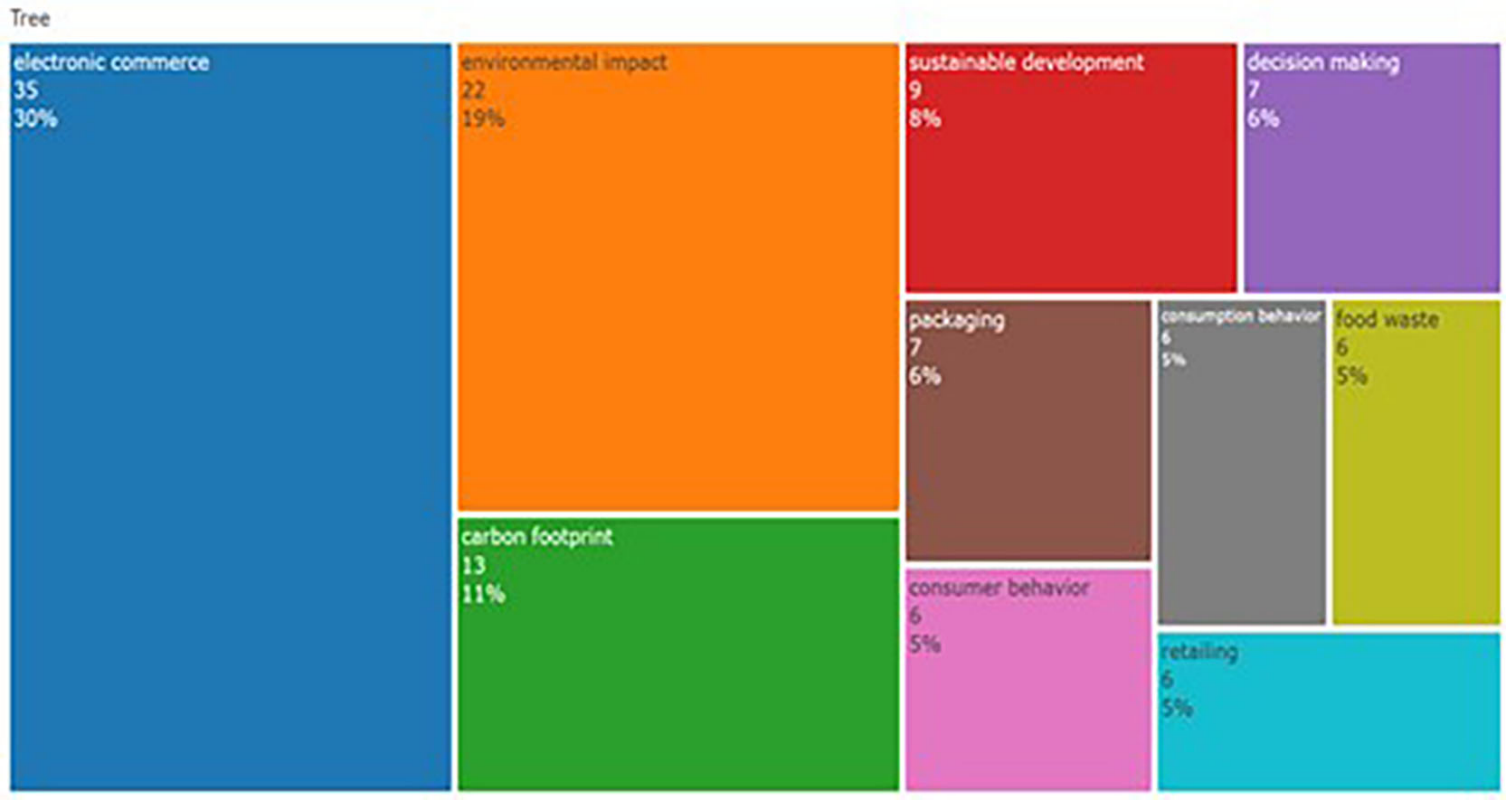
Tree map.

The tree map represents research topics from to 2014-2024 highlighting keywords related to e-commerce and sustainability. As can be seen in Figure 3, e-commerce has been a dominant theme, highlighting its importance and coverage in the last decade. The tree map also highlights research areas such as carbon footprint, “sustainable development,” and “packaging”, which further suggests the emphasis of researchers on sustainability in commerce. The emergence of keywords, decision making, and consumer behavior suggests that research attempts to reveal consumers’ interaction with e-commerce and sustainability efforts. Furthermore, the distribution of keywords reveals the interpretability of ‘sustainability in food’ as a research topic. The analysis also revealed recent and broad topics in the domain of sustainability of F&B.

### 3.3 Sankey diagram (Three field Plot)

**Figure 4.**
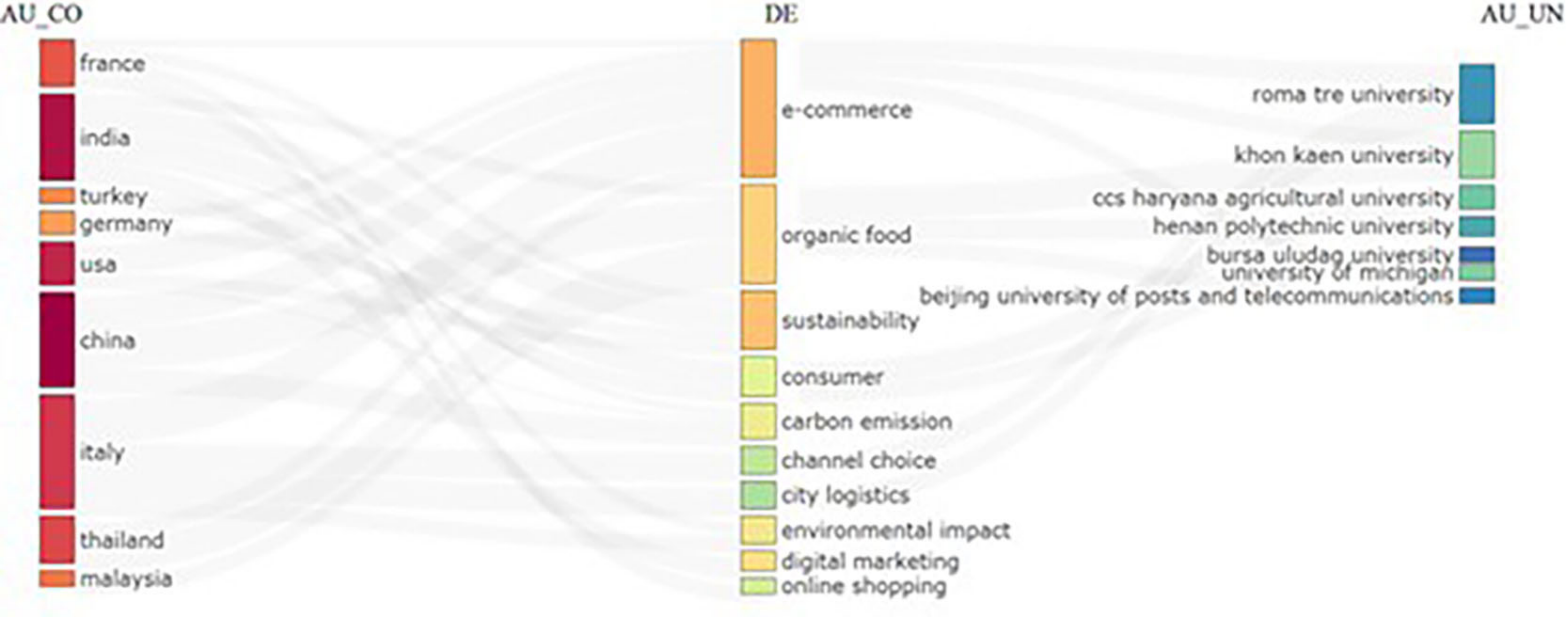
Three field plot.

The three field plots show research from multiple countries and their links with specific domains. The diagram further reveals that topics such as e-commerce and sustainability appeared as central themes ( Figure 4). The diagram shows that universities on the right-hand side have strong contributions to research in various domains. There is also a visible flow of research focus areas from different countries to universities, indicating possible international collaboration or research distribution.

The Sankey diagram lists the following attributes as: 1. AU_CO (Author Country): The Diagram also lists various countries, such as France, India, Turkey, Germany, USA, China, Italy, Thailand, and Malaysia, as countries represented by the authors. 2. DE (Domain/Topic): This reflects research topics such as e-commerce, organic food, sustainability, consumer behavior, carbon emissions, city logistics, environmental impact, and digital marketing as the focus areas of research. 3. AU_UN (Author University): List universities such as Roma Tre University, Khon Kaen University, CCS Haryana Agricultural University, Henan Polytechnic University, Bursa Uludağ University, Beijing University of Posts and Telecommunications, and the University of Michigan. It can be said that France is an active country researching this domain and their focused themes are ‘e-commerce’ and ‘organic food.’ India is the second leading country focusing on ‘sustainability,’ ‘digital marketing,’ ‘online shopping’ and ‘consumer.’ Major countries such as the USA, China, and Germany have comparatively less output linked with the ‘sustainability’ theme, which indicates a bright scope for further research. In terms of collaboration, countries such as China, India, Italy, and France depict multiple linkages, indicating strong collaboration across diverse research domains and universities.

### 3.4 Country-wise scientific production

**Figure 5.**
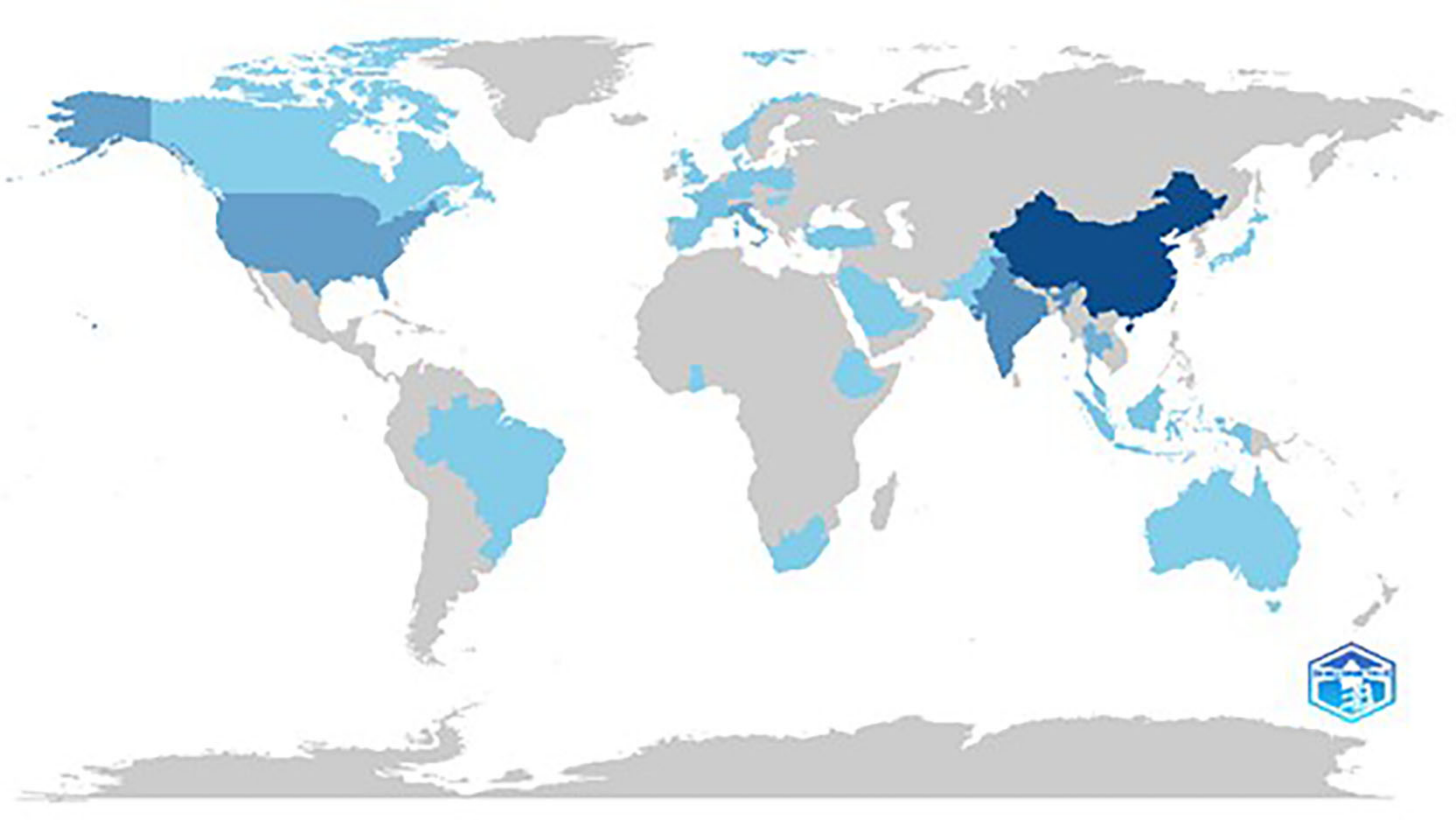
Country wise scientific production.

Figure 5 illustrates the scientific production in various countries based on the frequency of publishing articles. China and the USA are the leading contributors with the highest frequency of scientific publications, reflecting their dominant roles in global research output. This further indicates that the broad area of research carried out in these countries is covered under the theme of sustainable food marketing.

### 3.5 Most cited countries

**Figure 6.**
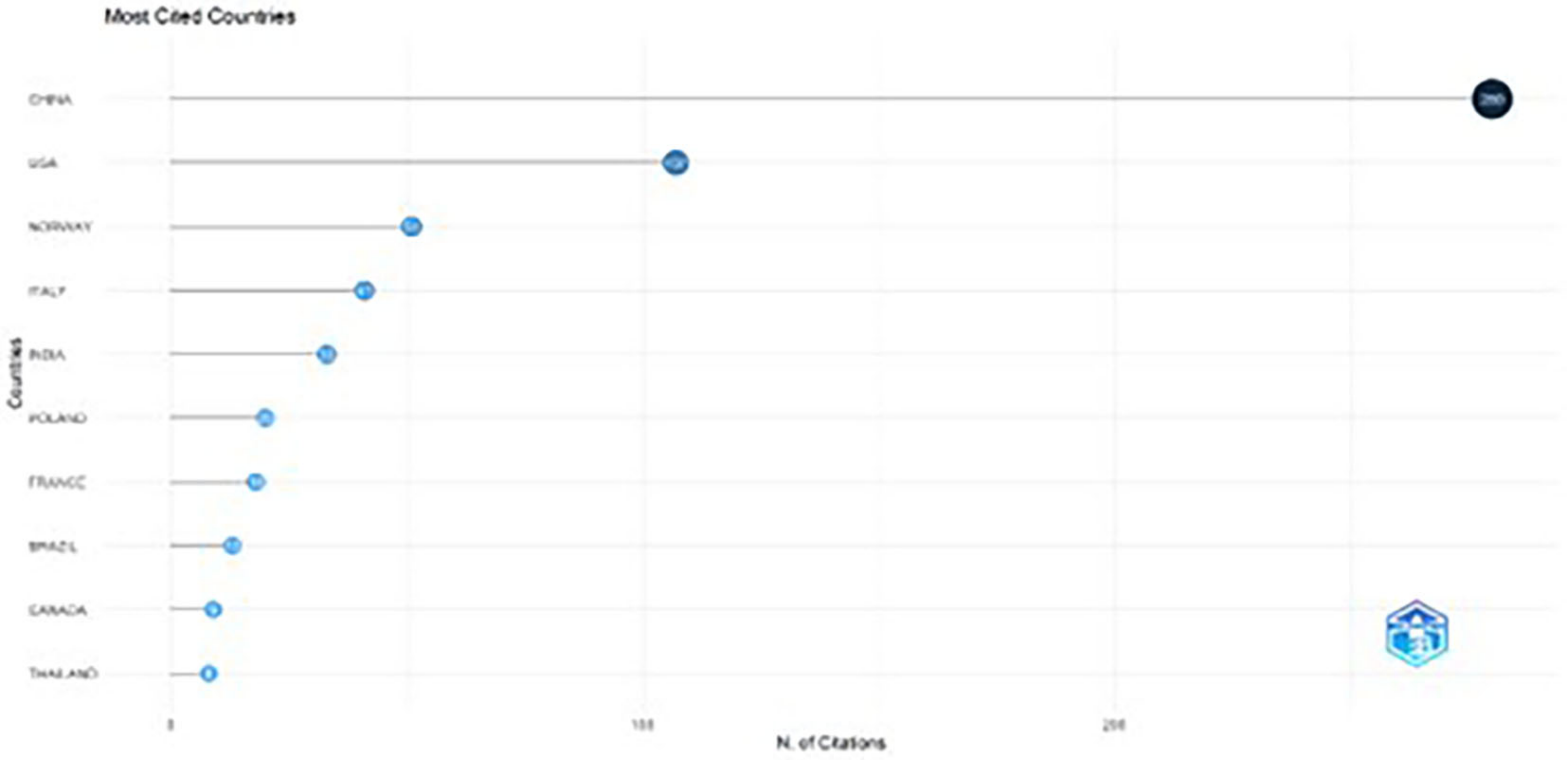
Most cited countries.

The line graph illustrates the post-article production trends covering citations. As shown in Figure 6, China had 280 citations, immediately followed by the USA with 107 citations. Norway ranks second with 51 citations, and Italy ranks fourth with 41 citations. Other countries, such as India (33), Poland (20), and France (18), show comparatively few citations. A possible reason for the low citation rates of papers from a few countries is a lack of confidence among readers. ^69^

### 3.6 CoWord network

**Figure 7.**
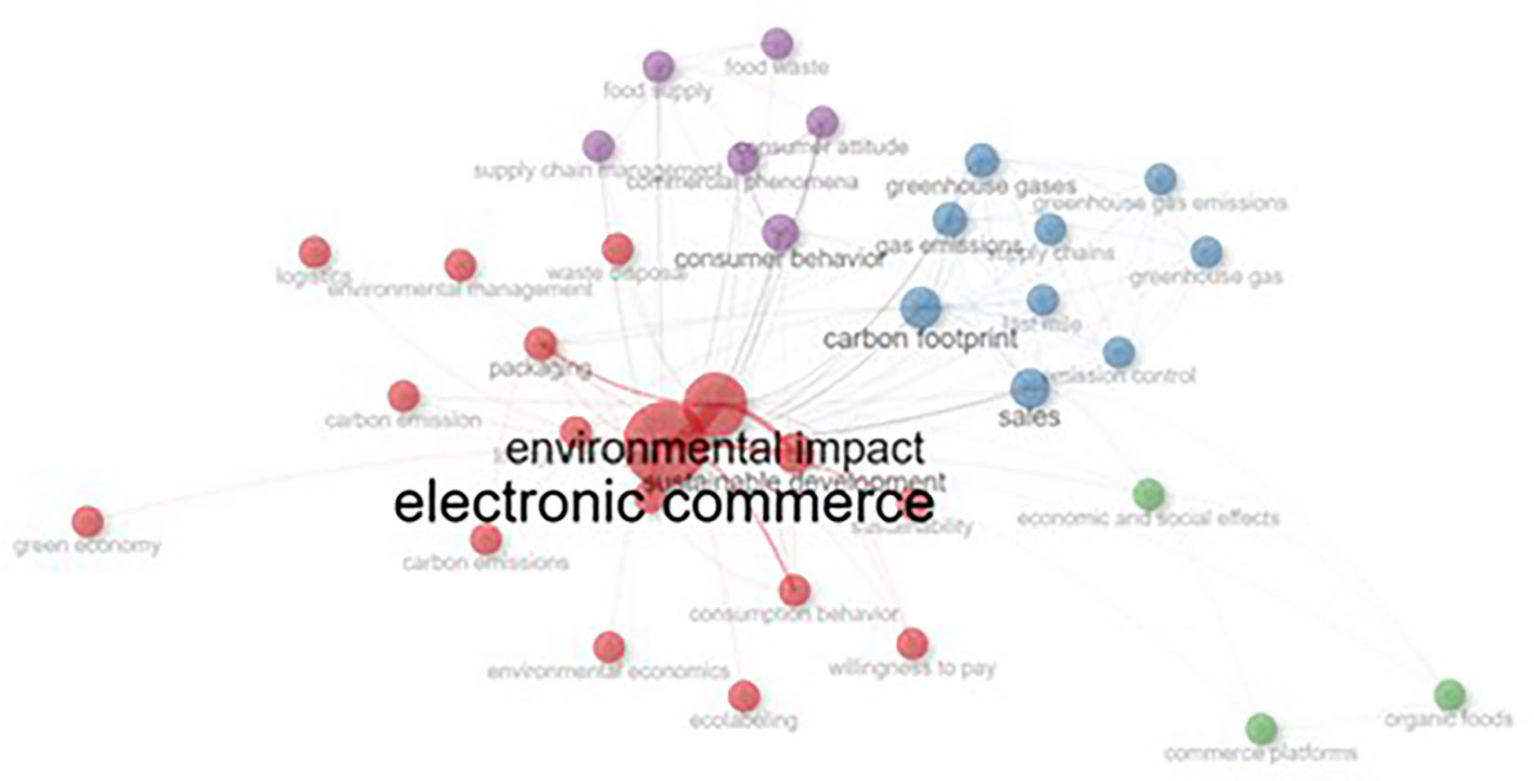
CoWord network.

The network visualization map in Figure 7 illustrates the relationships and co-occurrences of the various research topics. The central node, “electronic commerce,” is the most prominent, indicating its strong connection with other related areas such as “environmental impact”, “sustainable development,” “carbon footprint,” “sales” and “consumer behaviour.” The size of the nodes reflects the importance or frequency of the terms, whereas the thickness of the lines represents the strength of the connection between topics. Topics such as “greenhouse emissions,” “supply chain,” and “green economy” are clustered around green brands, showing their relevance to the field. Other smaller clusters, like “commercial phenomena” and “waste disposal,” are also present but less connected to the central theme.

We followed a manual clustering approach to evaluate the relevance of each set of studies. Manual screening was adopted over the algorithmic screening method to add a layer of expert judgment, which is otherwise difficult to establish. In addition, owing to the small number of studies and their nuanced outcomes, algorithmic methods might lack the necessary understanding. After the initial screening, we finalized a set of relevant articles. In the second step, researchers read all the papers to extract common areas of research, methodologies, and findings. Subsequently, categories were created to group similar articles. Within each identified cluster, we identified themes related to sustainability and digital marketing that connected the papers. Based on the commonalities in the identified themes and subthemes, we identified four clusters. As shown in Figure 7, there are four clearly visible clusters with the following colors: 1. Green, Blue, Purple, and Red. Based on these clusters, we identified the emerging research potential of sustainable branding and digital marketing in general.

## 4. Implications and research gaps

These findings make theoretical contributions to the field of sustainable digital marketing. This review advances existing understanding by mapping studies related to digital marketing, consumer decision-making, preferences, engagement, and digital channels using bibliometric techniques such as co-occurrence, citation analysis, three field plots, and emerging trends. Through the integration of these methods, we identified major thematic clusters that validate the interdisciplinary nature of digital channels, consumer behavior, economics, and consumer preferences. Using the 5W1H framework, this study devises a structured approach to understand the enablers of digital marketing strategies such as assessing brand impact, logistics, brand identity, and economics. Furthermore, this research presents a novel understanding of under-investigated areas in the broad domain of digital marketing and sustainability.

This study highlights the urgent need for novel understanding of sustainable F&B digital marketing strategies. Through an in-depth analysis of collaborations, publication output, citation count, and co-occurrence of research topics, this research aims to synthesize evidence on digital marketing strategies involving sustainable F&B. This review is an attempt to consolidate the findings in this area of research and devise meaningful strategies that are otherwise fragmented.

Descriptive data highlight the focus of the literature on digital marketing strategies for sustainable F&B brands over the last decade, with most articles published between 2020 and 2024. Publications from 2020 onward witnessed a significant rise due to high emphasis on sustainability and green marketing post the COVID-19 era. Notably, the most cited country, that is, China with 280 citations, leads the way with a huge margin, followed by the US with 107 citations. This highlights China’s research emphasis and contributions in this domain. In addition, the highly cited contributions published between 2014 and 2021 have especially focused on the purchase determinants, intentions, and behavior of consumers towards online purchases of sustainable foods. ^70– 74^ We also observed that most of these studies are quantitative in nature. Overall, the data suggest an increasing interest from researchers in determining strategies for sustainable F&B brands in the digital space.

The research questions (RQ1) framed to guide this study can be addressed by pointing out a notable rise in the number of studies covering this broad domain. In addition, studies on the strategies adopted by e-commerce platforms to market sustainable F&B are gaining attention. Overall, the analysis of studies reveals that as consumers tend to buy more from online platforms, digital strategies are also evolving with this trend. The use of advanced technologies such as AI is disrupting the marketplace, as marketers continue to leverage data and technology to optimize marketing. ^19^

The analysis revealed that the studies are guided by different perspectives covered by a broad area of research (RQ2). Based on the analysis, this study provides a strategic framework for brands to promote sustainable F&B through digital marketing channels. The results indicate a significant growth in research over the last decade, revealing collaboration and scientific potential. Furthermore, this review also points out an upward trend in the area of research, covering key keywords such as e-commerce, sustainability, and carbon footprints. To understand the influence of the four dominant strategic perspectives, we grouped them into four clusters based on impact: logistics, marketing, and economics. The clusters were identified based on the overall impression of the brand’s perception in the market.

Based on CoWord analysis, this study identified relationships and interactions between the topics researched and emerging research areas (RQ3). Interestingly, the investigation revealed that most of the research highlighted the perspectives of consumers; hence, existing research has not covered the strategic point of view. In addition, studies have measured behavior, experience, trust, and intentions, whereas the inside-out and outside-in perspectives of strategists are missing in the available literature. The perspectives linked with policy frameworks, incentives, or price regulation are few grey areas that can be covered in future research. A detailed description of the gray areas is provided in Table 1.

**Table 1.** Grey areas for future research.

Missing focus in existing studies	Future research areas	Priority (Theoretical/Managerial)
Over representation of China, US, EU and underrepresentation of global south	Geographic Coverage	Very High
Emotional branding perspectives, re-purchase and post-purchase analysis	Variation in Behavioural assessment	Medium
Artificial Intelligence and blockchain	Technological Growth	Very High
Market regulation, cost benefit analysis	Policy Factors	High
Ecolabelling, greenwashing, greenhushing in brands	Trust Building	Low

**Figure 8.**
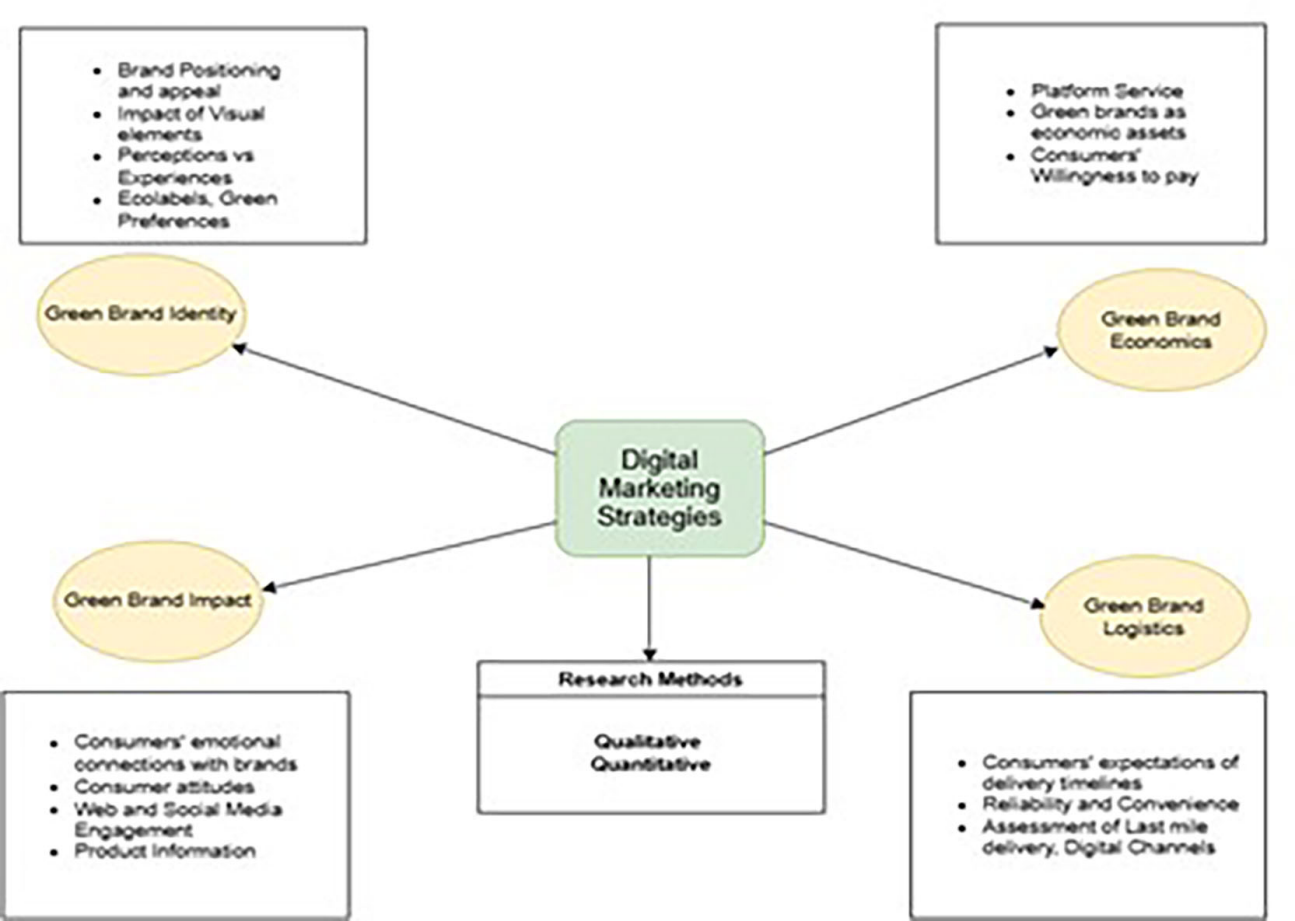
Areas for future research.

Based on the analysis, the following strategies can be suggested for future research. As illustrated in Figure 8, four clusters were identified. A description of resulting clusters is as under.

### 4.1 Green brand impact

This cluster centres at the intersection of factors including consumer emotions, attitudes, the alignment of buyer-supplier values, and its impact on the positioning of sustainable food or beverage brands. Other factors such as disclosures about brand governance and brand audits may impact consumers’ decision-making, as F&B require the highest level of quality standards. This cluster advances the existing understanding and engagement in green products. These attributes facilitate trust and social identity among stakeholders. ^75^ This cluster highlights growing academic and policy interest in establishing green attributes and product presentations on consumer trust. ^74, 76^ Future research should evaluate consumers’ emotional connections and their engagement in social media to determine their preferences. An evaluation of product information, digital marketing interactions, and their impact on purchase decisions could influence green branding in formulating digital marketing strategies. ^77^ Marketers can leverage emotional branding to trigger a response and incentivize purchases. ^78^ Further research can explore the construct across different cultural contexts to investigate the elements of branding that prompt a positive consumer response.

### 4.2 Green brand logistics

This cluster lies in the evolving domain of active interactions, including last mile delivery. ^79^ Digital marketing strategies in e-commerce logistics play an important role in determining e-commerce success. A digitized food supply chain can be used as a central theme to manage a sustainable food supply chain. ^80^ From a managerial perspective, incorporating sustainability into the supply chain requires the coordination of all stakeholders. Although digital marketing has transformed the marketplace, various hurdles continue to exist for the successful implementation of green logistics. ^81^ Overcoming barriers will enable F&B brands to create a positive brand image through long-term consistency, covering customer convenience, product availability, flexibility, and digital engagement. ^82, 83^ Future researchers should attempt to integrate sustainable approaches into both forward and reverse logistics operations with the aim of balancing the social, environmental, and economic dimensions. In addition, research can be conducted on the consumer acquisition process and the underlying factors to balance operational efficiency at the micro- and macro-levels.

### 4.3 Green brand identity

This cluster focuses on the significance of emerging labelling systems, such as ecolabels, green brand positioning, sentiments, and visual elements affecting food choices. ^84^ The cluster also includes a combination of studies on ecolabels, consumer preferences, and brand appeal. ^12, 85^ Studies within this cluster emphasize impulsive buying behavior and consumer sentiment. ^86, 87^ Food and its visual presentation are known to elicit emotions among consumers. ^88^ Marketers can devise strategies related to the visual appeal of F&B in gauging sensory expectations and hedonic evaluations among consumers. Furthermore, a focus on design cues can be linked to determine attention and affective, cognitive, and motivational reactions in buyers. ^89^ Future studies on the efficacy of visual design, including packaging, environment, graphical elements, and packaging size, should be considered. Additionally, elements such as the role of online displays and logos or labels can be determined by impacting F&B choices.

### 4.4 Green brand economics

This cluster integrates studies covering the determinants of organic online shopping, willingness to pay. ^13, 90^ Brand managers can effectively use emerging technologies such as platform services to forecast and drive demand to manage inventory efficiently. ^91^ In addition, practitioners can explore future possibilities associated with green brands by prioritizing green innovation and acquisition in F&B. ^92^ Leveraging green brands in the F&B industry as *strategic assets* can reinforce organizations’ sustainability goals by shaping trends within the marketplace. Future studies should capture the value aspects of the customer, including conversion rates and time taken to convert, in addition to gauging consumer willingness to purchase green F&B products. As e-commerce is evolving rapidly, brands need to adjust their strategies according to organizational goals.

**Figure 9.**
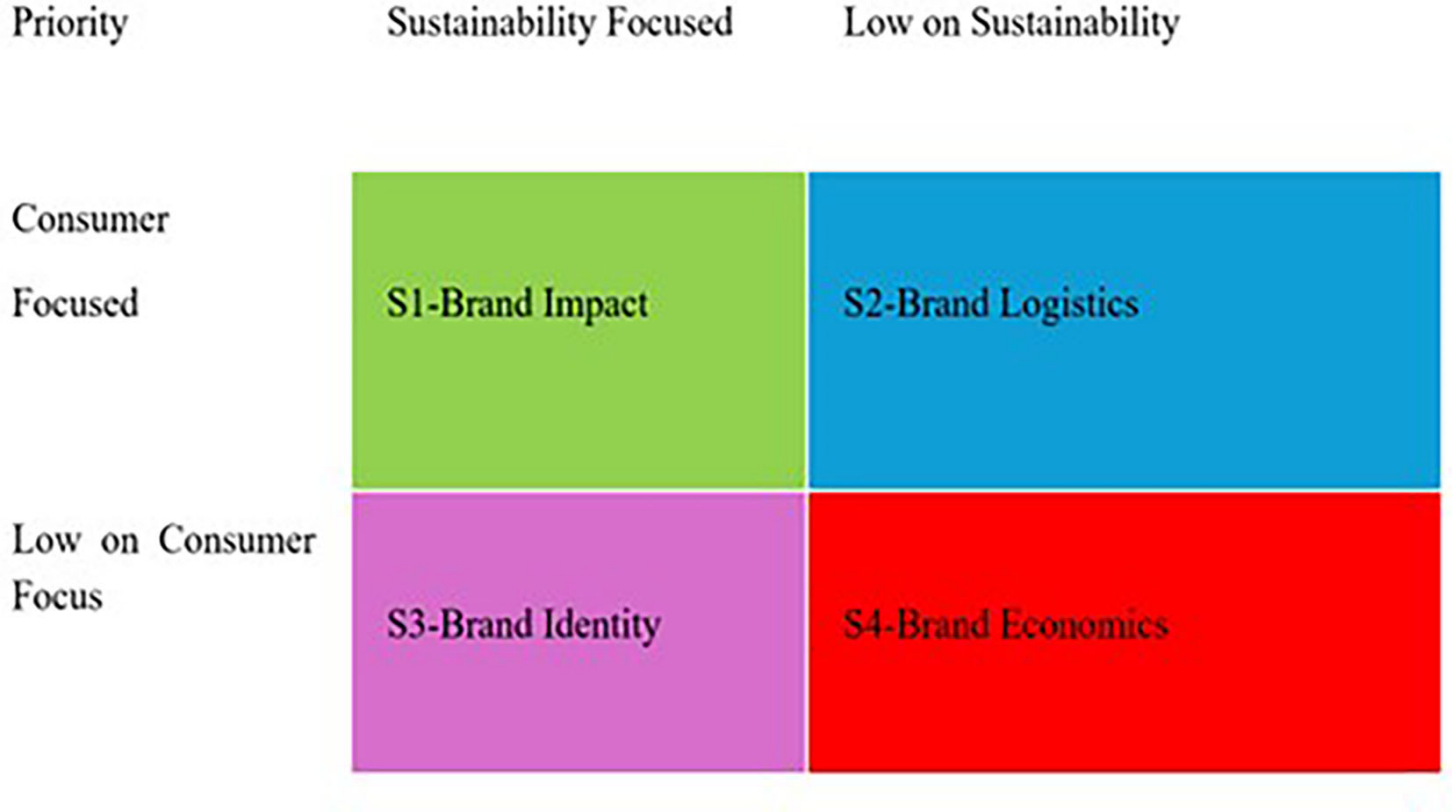
Strategy grid for brands.

From a strategic perspective, we formulated a strategy grid based on the clusters retrieved from a set of studies ( Figure 9). As can be seen in the four quadrants, brands can use the underlying strategies to devise ways forward. For ease of use and understanding, brand managers can select a set of priorities to obtain a desired strategy that can be implemented. Therefore, from a strategic point of view, this study adds value to emerging sustainable brands by setting their foot in the domain of digital marketing.

Current study provides meaningful insights to marketers and researchers. Marketers looking to develop brand-specific strategies can use the research findings by identifying their priorities. This will not only help the brands engage effectively with the customers but would also ensure the longevity of business profits . Additionally, researchers interested in sustainable brand strategies can use the findings to plan for studies on the identified topics by observing the research trends, as well as research gaps in this domain. Additionally, journal editorial boards can set standards for scholarly journals against these findings and advance guidelines for publishing research on the sustainability front.

## 5. Theoretical contributions/Advancements:

The review identifies clusters of variables that shape integrated marketing across different F&B categories. Based on the literature, we have identified several under-researched areas within each cluster and propose future research directions. In the context of modern-day marketing, organizations gather market intelligence that enables them to gain competitive advantage. This study advances the existing literature on brand identity ^93^ by proposing new areas of research that can assist organizations in refining their overall brand strategy. It contributes to the resource-based view by strengthening the argument that a firm’s competitive advantage is fundamentally derived from its unique resources and capabilities. ^94, 95^ The findings also enrich the resource orchestration framework ^96^ to direct specific managerial actions toward sustainable F&B brands. Therefore, examining the role of these resources and their impact on brand awareness, brand equity, and ultimate consumer behavior can offer valuable insights into devising strategies aligned with sustainable F&B brands. Given the relevance of sustainability and consumer-focused actions ( Figure 9), the findings suggest that in line with the evolution of resource-based logic, firms can leverage the competitive dynamics that lead to market advantage and improve performance. Thus, this review extends the resource-based view in the context of digital branding by emphasizing the relationship between strategic resources and firm performance.

This study adds a key contribution to the literature in the resource orchestration category by highlighting the importance of structuring a firm’s brand resource portfolio. It also argues that combining resources and transforming them into capabilities can lead to positive outcomes for digital brands. This study enriches the literature by reviewing scholarly research on digital marketing and sustainable F&B brands. The review sought to close the gap between what is already known in the domain of F&B marketing and what needs to be known in shaping strategies for brands operating in the digital sphere. Moreover, the present also study provides valuable insights for practitioners and researchers by providing a roadmap for the identification of future research areas. Being an underexplored domain in digital marketing, brands can utilize the outcomes of this study to influence the decision-making process of consumers by setting a priority embedded in the digital marketing space.

## 6. Conclusion, limitations and future research agenda

This study attempts to highlight the research on sustainable digital marketing to underline strategies for digital marketers. The review explored the Scopus database for this purpose and retrieved scholarly articles for analysis. This systematic review synthesizes evidence from 53 studies to affirm that in digital brand marketing, the best resource for a firm is its brand attributes, which can be applied to diverse marketing scenarios. Additionally, this study stresses the combination of strategic brand assets with a firm’s overall objectives to elevate business performance. This review offers valuable insights for practitioners by connecting traditional brand attributes with digital marketing, while also identifying prospects for future research.

Despite the application of well-validated processes, this study had several limitations. First, the database selected for article retrieval was only Scopus and did not include the Web of Science database, which limits the number of articles that were retrieved using keywords. In addition, the documents were explored using only a limited set of keywords; hence, other keywords that could have been relevant were not considered. Furthermore, the coverage of the articles determined was from 2014-2024, resulting in a limited number of research articles. This study did not use any validation through statistical techniques, which can lead to methodological bias in the interpretation of the findings. Also, the analysis of this study is subject to geographic bias, as the publications included are from specific countries which limit the comprehensive representation of the Global South. In future research, Digital Marketing innovation and the implementation of digital marketing strategies can be conducted. More specifically, future studies can be designed by considering the specific role of social media strategies for digital marketing and sustainability. A quantitative study probing digital brand attributes in F&B marketing and their impact on consumer decision making can provide detailed insights into strategic brand perspectives.

### Declaration on the use of AI statement

The authors confirmed that no generative Artificial Intelligence (AI) tools were used in the conceptualising this research or writing, data analysis, and interpretation of this study.

## Ethics approval and consent to participate

There was no requirement for ethical permission.

## Data Availability

This work contains the following underlying data: [Figshare]: Soodan, Vishal (2025). Digital Marketing Strategies for Sustainable Food and Beverage Brands: A Bibliometric Systematic Literature Review. figshare. Dataset. https://doi.org/10.6084/m9.figshare.30971752.v1. ^97^ Data are available under the terms of the Creative Commons Zero “No rights reserved” data waiver (CC0 1.0 Public domain dedication).

## References

[1] Weber H Sciubba JD : The Effect of Population Growth on the Environment: Evidence from European Regions. *Eur. J. Population.* 2019;35(2):379–402. 10.1007/s10680-018-9486-0 31105504 PMC6497702

[2] Hoa PX Xuan VN Thu NTP : Factors affecting carbon dioxide emissions for sustainable development goals – New insights into six asian developed countries. *Heliyon.* 2024;10(21):e39943. 10.1016/j.heliyon.2024.e39943 39553582 PMC11564035

[3] The Guardian environment pledge:2024. 2024 Nov 19 [cited 2025 Feb 2]. Reference Source

[4] White K Habib R Hardisty DJ : How to SHIFT Consumer Behaviors to be More Sustainable: A Literature Review and Guiding Framework. *J. Mark.* 2019;83(3):22–49.

[5] Dogaru L : Eco-Innovation and the Contribution of Companies to the Sustainable Development. *Procedia Manufacturing.* 2020;46:294–298.

[6] Achmad GN Yudaruddin R Nugroho BA : Government support, eco-regulation and eco-innovation adoption in SMEs: The mediating role of eco-environmental. *J. Open Innov.: Technol. Mark. Complex.* 2023;9(4):100158.

[7] Bajar RGCA Ong AKS German JD : Determining Sustainable Purchase Behavior for Green Products from Name-Brand Shops: A Gen Z Perspective in a Developing Country. *Sustainability.* 2024;16(9):3747.

[8] Moshood TD Nawanir G Mahmud F : Biodegradable plastic applications towards sustainability: A recent innovations in the green product. *Cleaner Engineering and Technology.* 2022;6:100404. 10.1016/j.clet.2022.100404

[9] Nascimento J Loureiro SMC : Mapping the sustainability branding field: emerging trends and future directions. *JPBM.* 2024;33(2):234–257.

[10] Bâra A Oprea SV Bucur C : Unraveling the Impact of Lockdowns on E-commerce: An Empirical Analysis of Google Analytics Data during 2019–2022. *JTAER.* 2023;18(3):1484–1510.

[11] Udayana AAGB Fatmawaty AS Makbul Y : Investigating the role of e-commerce application and digital marketing implementation on the financial and sustainability performance: An empirical study on Indonesian SMEs. 2023;8(1):167–178.

[12] Sharma AP : Consumers’ purchase behaviour and green marketing: A synthesis, review and agenda. *Int. J. Consum. Stud.* 2021;45(6):1217–1238.

[13] Irfan A Bryła P : Green marketing strategies for sustainable food and consumer behavior: A systematic literature review and future research agenda. *J. Clean. Prod.* 2025;486:144597.

[14] Gelgile HK Shukla A : Digital Marketing as an Enabler of Sustainable Food System: The Mediating Role of Relationship Marketing. *J. Int. Food Agribus. Mark.* 2024;36(1):93–102.

[15] Andriana R : The Influence of Digital Marketing and Brand Trust on Customer Loyalty Through Customer Satisfaction of Food and Beverage Companies. *IJESSM.* 2025;5(1):214–224.

[16] Maksi SJ Keller KL Dardis F : The food and beverage cues in digital marketing model: special considerations of social media, gaming, and livestreaming environments for food marketing and eating behavior research. *Front. Nutr.* 2024;10:1325265. 38384857 10.3389/fnut.2023.1325265 PMC10880034

[17] Chua XH Whitton C Vandevijvere S : Characterising the extent and nature of digital food and beverage marketing in Singapore: a descriptive study. *Public Health Nutr.* 2025;28(1):e14. 10.1017/S1368980024002428 PMC11822605 39663980

[18] Sarkis N Jabbour Al Maalouf N Al GS : The Power of Digital Engagement: Unveiling How Social Media Shapes Customer Responsiveness in the Food and Beverage Industry. *Administrative Sciences.* 2025;15(7):278.

[19] Driessen C Chung A Martino F : Contemporary digital marketing techniques used in unhealthy food campaigns targeting young people. *Appetite.* 2025;211:107989. 40185242 10.1016/j.appet.2025.107989

[20] Whitton C Wong YHM Lau J : Ecological momentary assessment of digital food and beverage marketing exposure and impact in young adults: A feasibility study. *Appetite.* 2024;197:107338. 38579981 10.1016/j.appet.2024.107338

[21] Dupuis R Musicus AA Edghill B : How TikTok Influencers Disclose Food and Beverage Brand Partnerships: Descriptive Study. *J. Med. Internet Res.* 2025;27:e60891. 10.2196/60891 40053812 PMC11909480

[22] Alotaibi IS : Examining the success factors of influencer marketing campaigns in the food and beverage industry: an empirical study. *MSAR.* 2025 June 10 [cited 2025 Oct 28]. 10.1108/MSAR-12-2024-0223/full/html

[23] Boyland E Backholer K Potvin Kent M : Unhealthy Food and Beverage Marketing to Children in the Digital Age: Global Research and Policy Challenges and Priorities. *Annu. Rev. Nutr.* 2024;44(1):471–497. 10.1146/annurev-nutr-062322-014102 38631811

[24] Potvin Kent M Pritchard M Mulligan C : Normalizing junk food: The frequency and reach of posts related to food and beverage brands on social media. Duarte Batista L , editor. *PLOS Digit Health.* 2024 Oct 31;3(10):e0000630. 10.1371/journal.pdig.0000630 39480749 PMC11527147

[25] Frost H Te Morenga L Mackay S : Impact of unhealthy food/drink marketing exposure to children in New Zealand: a systematic narrative review. *Health Promot. Int.* 2025;40(2):daaf021. 10.1093/heapro/daaf021 40177787 PMC11965983

[26] Jia C Cai Y Yu YT : 5W+1H pattern: A perspective of systematic mapping studies and a case study on cloud software testing. *J. Syst. Softw.* 2016;116:206–219. 10.1016/j.jss.2015.01.058

[27] Paliwal V Chandra S Sharma S : Blockchain Technology for Sustainable Supply Chain Management: A Systematic Literature Review and a Classification Framework. *Sustainability.* 2020;12(18):7638.

[28] O’Donnell M Collier M Pineda-Pinto M : Redefining co-design for social-ecological research and practice: A systematic literature review. *Environ. Sci. Pol.* 2025;164:103998.

[29] Lisboa HM Pasquali MB Dos Anjos AI : Innovative and Sustainable Food Preservation Techniques: Enhancing Food Quality, Safety, and Environmental Sustainability. *Sustainability.* 2024 Sept 21;16(18):8223. 10.3390/su16188223

[30] Hobbs PR : Paper Presented at International Workshop on Increasing Wheat Yield Potential, Cimmyt, Obregon, Mexico, 20–24 March 2006. Conservation agriculture: what is it and why is it important for future sustainable food production? *J. Agric. Sci.* 2007;145(2):127–137. 10.1017/S0021859607006892

[31] McCarthy BL : Sustainable food systems in Northern Queensland. *Journal of Economic & Social Policy.* 2014;16(1):70–89.

[32] Henderson JC : Food as a Tourism Resource: A View from Singapore. *Tour. Recreat. Res.* 2004;29(3):69–74.

[33] Hsu FC Robinson RNS Scott N : Traditional food consumption behaviour: the case of Taiwan. *Tour. Recreat. Res.* 2018;43(4):456–469.

[34] Sobal J : Food System Channels, Health, and Illness. Shostak S , editor. *Advances in Medical Sociology.* Emerald Publishing Limited;2017 [cited 2025 Oct 28]; pp.3–25. 10.1108/S1057-629020170000018001 Reference Source

[35] Sellitto MA Vial LAM Viegas CV : Critical success factors in Short Food Supply Chains: Case studies with milk and dairy producers from Italy and Brazil. *J. Clean. Prod.* 2018;170:1361–1368.

[36] Hoek AC Luning PA Weijzen P : Replacement of meat by meat substitutes. A survey on person- and product-related factors in consumer acceptance. *Appetite.* 2011;56(3):662–673. 10.1016/j.appet.2011.02.001 21315123

[37] Lee YY Gan CL Liew TW : Rationality and impulse buying: Is your emotion a part of the equation? *Comput. Hum. Behav. Rep.* 2023;12:100337. 10.1016/j.chbr.2023.100337

[38] Smale MC Fox JD Fox AK : When being smart trumps AI: An exploration into consumer preferences for smart vs. AI-powered products. *Computers in Human Behavior.* 2024;161:108405.

[39] Torossian R : The Future of Food Marketing: How Digital Tools Are Shaping the Industry. *Medium.* 2024 [cited 2025 Dec 16]. Reference Source

[40] Sloan P Legrand W Hindley C : *The Routledge Handbook of Sustainable Food and Gastronomy.* Bungay: Routledge; Reference Source

[41] Dabas S Sharma S Manaktola K : Adoption of digital marketing tools in independent businesses: experiences of restaurant entrepreneurs in India and United Kingdom. *WHATT.* 2021;13(2):214–235.

[42] Constantinides E : Foundations of Social Media Marketing. *Procedia. Soc. Behav. Sci.* 2014 Aug;148:40–57. 10.1016/j.sbspro.2014.07.016

[43] Zehir C Şahin A Kitapçı H : The Effects of Brand Communication and Service Quality In Building Brand Loyalty Through Brand Trust; The Empirical Research On Global Brands. *Procedia. Soc. Behav. Sci.* 2011;24:1218–1231.

[44] Kannan PK Li H : “Alice.” Digital marketing: A framework, review and research agenda. *Int. J. Res. Mark.* 2017;34(1):22–45.

[45] Dossena C Mochi F Bissola R : Restaurants and social media: rethinking organizational capabilities and individual competencies. *JTF.* 2021;7(1):20–39.

[46] Fraccastoro S Gabrielsson M Pullins EB : The integrated use of social media, digital, and traditional communication tools in the B2B sales process of international SMEs. *Int. Bus. Rev.* 2021;30(4):101776.

[47] Saura JR Palos-Sánchez P Cerdá Suárez LM : Understanding the Digital Marketing Environment with KPIs and Web Analytics. *Future Internet.* 2017;9(4):76.

[48] Dwivedi YK Ismagilova E Hughes DL : Setting the future of digital and social media marketing research: Perspectives and research propositions. *Int. J. Inf. Manag.* 2021;59:102168.

[49] Berthon PR Pitt LF Plangger K : Marketing meets Web 2.0, social media, and creative consumers: Implications for international marketing strategy. *Bus. Horiz.* 2012;55(3):261–271.

[50] Lepkowska-White E Parsons A Berg W : Social media marketing management: an application to small restaurants in the US. *IJCTHR.* 2019;13(3):321–345.

[51] Palmié M Miehé L Oghazi P : The evolution of the digital service ecosystem and digital business model innovation in retail: The emergence of meta-ecosystems and the value of physical interactions. *Technol. Forecast. Soc. Chang.* 2022;177:121496.

[52] Marzi G Balzano M Caputo A : Guidelines for Bibliometric-Systematic Literature Reviews: 10 steps to combine analysis, synthesis and theory development. *Int. J. Manag. Rev.* 2025;27:81–103. 10.1111/ijmr.12381

[53] Khan T : A promising year for e-commerce sector. *Business Today.* 2014 Dec 24 [cited 2025 Jan 12]. Reference Source

[54] *Global Powers of Retailing 2014 Retail Beyond begins.* Australia:2014 Jan [cited 2025 Feb 18]. file:///C:/Users/vishal.soodan/Downloads/Deloitte-lideres-comercio-mundial-ReasonWhy.es_.pdf.

[55] Carrera-Rivera A Ochoa W Larrinaga F : How-to conduct a systematic literature review: A quick guide for computer science research. *MethodsX.* 2022;9:101895. 36405369 10.1016/j.mex.2022.101895 PMC9672331

[56] Breslin D Gatrell C : Theorizing Through Literature Reviews: The Miner-Prospector Continuum. *Organ. Res. Methods.* 2023;26(1):139–167.

[57] Schoombee L : Why Scopus is essential for your literature review.

[58] Page MJ McKenzie JE Bossuyt PM : The PRISMA 2020 statement: An updated guideline for reporting systematic reviews. *Int. J. Surg.* 2021;88:105906. 33789826 10.1016/j.ijsu.2021.105906

[59] Ibidunni AS Ufua DE Opute AP : Linking disruptive innovation to sustainable entrepreneurship within the context of small and medium firms: A focus on Nigeria. *Afr. J. Sci. Technol. Innov. Dev.* 2022;14(6):1591–1607.

[60] Kao PJ Dacko S Hu Y : Unraveling the complexity of radical service innovation: A systematic review, integrative framework, and research roadmap. *J. Bus. Res.* 2026;202:115754. 10.1016/j.jbusres.2025.115754

[61] Martín-Martín A Thelwall M Orduna-Malea E : Google Scholar, Microsoft Academic, Scopus, Dimensions, Web of Science, and OpenCitations’ COCI: a multidisciplinary comparison of coverage via citations. *Scientometrics.* 2021;126(1):871–906. 10.1007/s11192-020-03690-4 32981987 PMC7505221

[62] Pizzi S Caputo A Corvino A : Management research and the UN sustainable development goals (SDGs): A bibliometric investigation and systematic review. *J. Clean. Prod.* 2020;276:124033.

[63] Deng Y Yang Q Hao C : Combined lifestyle factors and metabolic syndrome risk: a systematic review and meta-analysis. *Int. J. Obes.* 2025;49(2):226–236. 10.1038/s41366-024-01671-8 39516361

[64] Marzi G Balzano M Marchiori D : K-Alpha Calculator–Krippendorff’s Alpha Calculator: A user-friendly tool for computing Krippendorff’s Alpha inter-rater reliability coefficient. *MethodsX.* 2024;12:102545. 39669968 10.1016/j.mex.2023.102545 PMC11636850

[65] Drucker AM Fleming P Chan AW : Research Techniques Made Simple: Assessing Risk of Bias in Systematic Reviews. *J. Invest. Dermatol.* 2016;136(11):e109–e114. 10.1016/j.jid.2016.08.021 27772550

[66] Hendricks S Mwapwele SD : A systematic literature review on the factors influencing e-commerce adoption in developing countries. *Data and Information Management.* 2024;8(1):100045.

[67] Templier M Paré G : A Framework for Guiding and Evaluating Literature Reviews. *CAIS.* 2015 [cited 2025 Oct 28];37. 10.17705/1CAIS.03706 Reference Source

[68] Uttley L Quintana DS Montgomery P : The problems with systematic reviews: a living systematic review. *J. Clin. Epidemiol.* 2023;156:30–41. 10.1016/j.jclinepi.2023.01.011 36796736

[69] Seifert R Hassan W : Country-specific citation disparities in Naunyn–Schmiedeberg’s Archives of Pharmacology from 2001 to 2024. *Naunyn Schmiedeberg’s Arch. Pharmacol.* 2025 Aug 14 [cited 2025 Oct 28]. 10.1007/s00210-025-04499-9 40810795 PMC12901205

[70] Cavallo C Sacchi G Carfora V : Resilience effects in food consumption behaviour at the time of Covid-19: perspectives from Italy. *Heliyon.* 2020;6(12):e05676. 10.1016/j.heliyon.2020.e05676 33313439 PMC7722488

[71] Lin J Li T Guo J : Factors influencing consumers’ continuous purchase intention on fresh food e-commerce platforms: An organic foods-centric empirical investigation. *Electron. Commer. Res. Appl.* 2021;50:101103.

[72] Rong-Da LA : Enthusiastically consuming organic food: An analysis of the online organic food purchasing behaviors of consumers with different food-related lifestyles. *Internet Res.* 2014;24(5):587–607.

[73] Śmiglak-Krajewska M Wojciechowska-Solis J Viti D : Consumers’ Purchasing Intentions on the Legume Market as Evidence of Sustainable Behaviour. *Agriculture.* 2020;10(10):424.

[74] Yue L Liu Y Wei X : Influence of online product presentation on consumers’ trust in organic food: A mediated moderation model. *BFJ.* 2017;119(12):2724–2739.

[75] Song Z Hu M Leng M : Exploring the Effects of Low-Carbon Labels on Purchase Intentions for Green Agricultural Products. *Sustainability (Switzerland).* 2024;16(17). Reference Source

[76] Ma Z Chen J Tian G : Regulations on the corporate social irresponsibility in the supply chain under the multiparty game: Taking China’s organic food supply chain as an example. *J. Clean. Prod.* 2021;317:128459.

[77] Armutcu B Ramadani V Zeqiri J : The role of social media in consumers’ intentions to buy green food: evidence from Türkiye. *BFJ.* 2024;126(5):1923–1940.

[78] Arens WF Weigold MF : *Contemporary advertising and integrated marketing communications.* New York, NY: McGraw-Hill; Sixteenth ed. 2021;702.

[79] Gruzauskas V Burinskiene A Krisciunas A : Application of Information-Sharing for Resilient and Sustainable Food Delivery in Last-Mile Logistics. *Mathematics.* 2023;11(2):303.

[80] Ekren BY Mangla SK Turhanlar EE : Lateral inventory share-based models for IoT-enabled E-commerce sustainable food supply networks. *Comput. Oper. Res.* 2021;130:105237.

[81] Panghal A Akhila P Vern P : Adoption barriers to green logistics in the Indian food industry: A circular economy perspective. *Int. Soc. Sci. J.* 2024;74(252):519–538.

[82] Ahn H : Unrevealing Voice Search Behaviors: Technology Acceptance Model Meets Anthropomorphism in Understanding Consumer Psychology in the U.S. Market. *Sustainability.* 2023;15(23):16455. 10.3390/su152316455

[83] Fatorachian H Arboleda E Linh TT : Digitalisation and customer engagement in fast-food SMEs: enhancing brand presence through social media. *Cogent Bus. Manag.* 2025;12(1):2508927.

[84] Yadav E Goyal M Ghalawat S : Factors Influencing Consumer Choices and Organic Food Production in Haryana: Insights from Farmers. *IJEE.* 2024;60(4):90–94.

[85] Hjelmar U : Consumers’ purchase of organic food products. A matter of convenience and reflexive practices. *Appetite.* 2011;56(2):336–344. 10.1016/j.appet.2010.12.019 21192997

[86] Kumar V Sindhwani R Zhang JZ : Optimizing short food supply chains through understanding consumer preferences for organic foods via e-commerce platforms and last-mile logistics. *BFJ.* 2025;127(5):1788–1809.

[87] Li L : Analysis of e-commerce customers’ shopping behavior based on data mining and machine learning. *Soft. Comput.* 2023 July 10 [cited 2025 May 6]. 10.1007/s00500-023-08903-5

[88] Berčík J Rusková A Predanócyová K : Visual presentation of food can influence appetite and emotions. *Cogent Food & Agriculture.* 2025 31;11(1):2575286. 10.1080/23311932.2025.2575286

[89] Vermeir I Roose G : Visual Design Cues Impacting Food Choice: A Review and Future Research Agenda. *Foods.* 2020;9(10):1495. 33086720 10.3390/foods9101495 PMC7589873

[90] Bryła P : Organic food online shopping in Poland. *BFJ.* 2018;120(5):1015–1027.

[91] Ganapathy V Gupta C Arora R : Digital Transformation of Grocery Delivery Services in India: Understanding the Past, Present, and Future. Garg A , editor. *Advances in Business Strategy and Competitive Advantage.* IGI Global;2024 [cited 2025 Dec 15]; pp.1–22. 10.4018/979-8-3693-7683-6.ch001

[92] Wei Y Pujari D : Drivers of green innovation and green acquisition: empirical evidence from the food and beverage industry. *JBIM.* 2025;40(1):101–115.

[93] Barney J : Firm Resources and Sustained Competitive Advantage. *J. Manag.* 1991;17(1):99–120.

[94] Peteraf MA : The cornerstones of competitive advantage: A resource-based view. *Strateg. Manag. J.* 1993;14(3):179–191.

[95] Wernerfelt B : A resource-based view of the firm. *Strateg. Manag. J.* 1984;5(2):171–180.

[96] D’Oria L Crook TR Ketchen DJ : The Evolution of Resource-Based Inquiry: A Review and Meta-Analytic Integration of the Strategic Resources–Actions–Performance Pathway. *J. Manag.* 2021;47(6):1383–1429.

[97] Soodan V : Digital Marketing Strategies for Sustainable Food and Beverage Brands: A Bibliometric Systematic Literature Review.Dataset. *figshare.* 2025. 10.6084/m9.figshare.30971752.v1 PMC12865291 41640733

