# Supplementary material for: Digital Marketing Strategies for Sustainable Food and Beverage Brands: A Bibliometric Systematic Literature Review
Source: F1000Res. 2026 Feb 20;15:27. Originally published 2026 Jan 7. [Version 2] doi: 10.12688/f1000research.176435.2 (PMC12865291; doi:10.12688/f1000research.176435.2)
Supplement: Peer review history

# Reviewer report: Reviewer response for version 1

**DOI:** 10.5256/f1000research.194489.r451379

**Published:** 2026-02-05

**Authors:** Lokesh Jasrai

**Affiliations:** 1 Lovely Professional University, Phagwara, Punjab, India

The topic is timely and relevant; however, the manuscript should more explicitly justify the **specific need to focus on Food and Beverage brands**. Given the sector’s unique characteristics—such as high environmental impact, frequent consumer touchpoints, perishable products, regulatory scrutiny, and increasing consumer sensitivity toward health and sustainability—the authors should clearly articulate why digital marketing strategies for sustainability in the F&B context warrant a dedicated bibliometric investigation rather than being subsumed under general sustainable branding or digital marketing literature.

Are the rationale for, and objectives of, the Systematic Review clearly stated?

Yes

Is the statistical analysis and its interpretation appropriate?

Not applicable

If this is a Living Systematic Review, is the ‘living’ method appropriate and is the search schedule clearly defined and justified? (‘Living Systematic Review’ or a variation of this term should be included in the title.)

Yes

Are sufficient details of the methods and analysis provided to allow replication by others?

Yes

Are the conclusions drawn adequately supported by the results presented in the review?

Yes

Reviewer Expertise:

1. QUALITATIVE RESEARCH- ATLAS.TI, NVIVO OR MAXQDA, 2. QUANTITATIVE ANALYSIS, 3. PLS-SEM BASED ANALYSIS, 4. MARKETING AND BUSINESS STUDIES

I confirm that I have read this submission and believe that I have an appropriate level of expertise to confirm that it is of an acceptable scientific standard.

# Response

**Published:** 2026-02-14

**Authors:** Vishal Soodan

**Affiliations:** Commerce, Manipal Academy of Higher Education, Manipal, Karnataka, India

Thank you so much for the review. We have added a paragraph covering justification for the point raised.

# Reviewer report: Reviewer response for version 1

**DOI:** 10.5256/f1000research.194489.r451387

**Published:** 2026-02-02

**Authors:** Meylani Tuti

**Affiliations:** 1 Universitas Asa Indonesia, Jakarta, Indonesia

To qualify as a Living Systematic Review, a manuscript must specify a clear update frequency (e.g., every 6 months or quarterly) and its rationale, describe any ongoing search automation or monitoring methods, and explain how new evidence will be filtered, integrated, and versioned so that the paper can be categorized as ongoing.

 the data used is only sourced from a database (Scopus) with a limited time period (2014–2024), thus potentially causing selection bias and not fully representing the entire global publication with a relatively small number of articles, namely 53 articles. With a descriptive mapping approach, this creates limitations in critical synthesis and the theory used. The author also did not conduct validation with advanced statistical techniques, which will cause bias in addition to geographic bias because the publications used come from certain countries and therefore are less able to represent the Global South region which is the area of ​​this study.

Are the rationale for, and objectives of, the Systematic Review clearly stated?

Yes

Is the statistical analysis and its interpretation appropriate?

Yes

If this is a Living Systematic Review, is the ‘living’ method appropriate and is the search schedule clearly defined and justified? (‘Living Systematic Review’ or a variation of this term should be included in the title.)

No

Are sufficient details of the methods and analysis provided to allow replication by others?

Yes

Are the conclusions drawn adequately supported by the results presented in the review?

Yes

Reviewer Expertise:

the data used is only sourced from a database (Scopus) with a limited time period (2014–2024), thus potentially causing selection bias and not fully representing the entire global publication with a relatively small number of articles, namely 53 articles. With a descriptive mapping approach, this creates limitations in critical synthesis and the theory used. The author also did not conduct validation with advanced statistical techniques, which will cause bias in addition to geographic bias because the publications used come from certain countries and therefore are less able to represent the Global South region which is the area of ​​this study.

I confirm that I have read this submission and believe that I have an appropriate level of expertise to confirm that it is of an acceptable scientific standard.

# Response

**Published:** 2026-02-14

**Authors:** Vishal Soodan

**Affiliations:** Commerce, Manipal Academy of Higher Education, Manipal, Karnataka, India

Thank you for the review. We have added these points as limitations in our study.

# Reviewer report: Reviewer response for version 1

**DOI:** 10.5256/f1000research.194489.r451380

**Published:** 2026-01-22

**Authors:** Praveen Gujjar

**Affiliations:** 1 JAIN (Deemed-to-be University), Bengaluru, Karnataka, India

The study is well-motivated and effectively captures the increasing importance of environmentally conscious consumer behavior in the digital marketing landscape. The analysis of 53 Scopus-indexed articles published between 2014 and 2024 provides a comprehensive overview of the conceptual structure and evolution of research in sustainable digital marketing. The paper successfully identifies emerging research streams and underexplored dimensions of sustainable digital marketing (SDM) within the food and beverage sector, thereby addressing an important research gap.

Are the rationale for, and objectives of, the Systematic Review clearly stated?

Yes

Is the statistical analysis and its interpretation appropriate?

Yes

If this is a Living Systematic Review, is the ‘living’ method appropriate and is the search schedule clearly defined and justified? (‘Living Systematic Review’ or a variation of this term should be included in the title.)

Not applicable

Are sufficient details of the methods and analysis provided to allow replication by others?

Yes

Are the conclusions drawn adequately supported by the results presented in the review?

Yes

Reviewer Expertise:

Sentiment analysis, Digital marketing

I confirm that I have read this submission and believe that I have an appropriate level of expertise to confirm that it is of an acceptable scientific standard.

## References

[1] : Modeling Customers' Intention to Use E-Wallet in a Developing Nation. *Journal of Electronic Commerce in Organizations* .2020;18(1) : 10.4018/JECO.2020010105 89-114 10.4018/JECO.2020010105
